# Treatment of Pyorrhœa Alveolaris

**Published:** 1896-12

**Authors:** 


### Treatment of Pyorrhœa Alveolaris.

Local anæsthesia; thorough removal of all deposits and diseased process; lactic acid as a solvent; trikresol (one-half of 1 percent solution) as an antiseptic wash; resorcin to produce adhesive inflammation. Pack large pockets of iodoform gauze. One treatment is all that is necessary in ninety-nine out of one hundred cases.—R. H. COOL, *in Pacific Stomatological Gazette.*

